# The role of microscopic properties on cortical bone strength of femoral neck

**DOI:** 10.1186/s12891-023-06248-6

**Published:** 2023-02-20

**Authors:** Ning Xia, Yun Cai, Qianhua Kan, Jian Xiao, Lin Cui, Jiangjun Zhou, Wei Xu, Da Liu

**Affiliations:** 1 Department of Orthopedics, The General Hospital of Western Theater Command, Chengdu, 610083 China; 2 Department of Critical Care Medicine, The Second Affiliated Hospital of Hainan Medical University, Haikou, 570311 China; 3 School of Mechanics and Aerospace Engineering, Southwest Jiaotong University, Chengdu, 611756 China; 4 Department of Endocrinology, The General Hospital of Western Theater Command, Chengdu, 610083 China; 5 Department of Orthopedic, The 908Th Hospital of Joint Logistic Support Force of PLA, Nanchang, 330001 China; 6 Trauma Center, The General Hospital of Western Theater Command, Chengdu, 610083 China

**Keywords:** Osteoporosis, Femoral neck, Cortical bone, Micro—structure, Micro—mechanics, Microscopic composition, Bone strength

## Abstract

**Background:**

Femoral neck fractures are serious consequence of osteoporosis (OP), numbers of people are working on the micro—mechanisms of femoral neck fractures. This study aims to investigate the role and weight of microscopic properties on femoral neck maximum load (L_max_), funding the indicator which effects L_max_ most.

**Methods:**

A total of 115 patients were recruited from January 2018 to December 2020. Femoral neck samples were collected during the total hip replacement surgery. Femoral neck Lmax, micro—structure, micro—mechanical properties, micro—chemical composition were all measured and analyzed. Multiple linear regression analyses were performed to identify significant factors that affected the femoral neck L_max_.

**Results:**

The L_max_, cortical bone mineral density (cBMD), cortical bone thickness (Ct. Th), elastic modulus, hardness and collagen cross—linking ratio were all significantly decreased, whereas other parameters were significantly increased during the progression of OP (*P* < 0.05). In micro—mechanical properties, elastic modulus has the strongest correlation with L_max_ (*P *< 0.05). The cBMD has the strongest association with L_max_ in micro—structure (*P* < 0.05). In micro—chemical composition, crystal size has the strongest correlation with L_max_ (*P* < 0.05). Multiple linear regression analysis showed that elastic modulus was most strongly related to L_max_ (β = 0.920, *P* = 0.000).

**Conclusions:**

Compared with other parameters, elastic modulus has the greatest influence on L_max_. Evaluation of microscopic parameters on femoral neck cortical bone can clarify the effects of microscopic properties on L_max_, providing a theoretical basis for the femoral neck OP and fragility fractures.

## Introduction

Osteoporosis (OP) is a metabolic skeletal disorder characterized by decreased bone mass and micro—structural destruction of bone tissue, which ultimately leads to increased bone fragility and fracture risk [1]. Osteoporotic fracture can lead to serious consequences, including disability, loss of capacity for independent living, higher healthcare costs, and excess mortality [2–4].

Osteoporotic fractures have become a public health problem [5, 6]. Bone mineral density (BMD) is an important indicator for diagnosing OP and predicting the risk of osteoporotic fractures [7]. At present, dual—energy X—ray absorptiometry (DEXA) is the reference standard and the most widely used method to assess BMD [6]. Due to the rapid detection, wide detection range, and non-invasiveness, DEXA has been widely used in clinical practice. However, some studies have revealed that some non—vertebral fractures occur in individuals without OP (T-score > -2.5), fracture risk cannot be accounted for by BMD alone [8, 9]. Bone strength is one of the important factors that predict clinical fracture risk. To evaluate the bone strength more comprehensively, bone mass (bone mineral content) and bone quality (bone micro—structure, bone geometry, bone turnover status and bone micro—damage) were assessed [10–12].

Hip fracture is one of the severe bone fragile fractures among osteoporotic injuries [13, 14]. Among them, femoral neck fracture is the common type, accounting for approximately 50% of all hip fractures [13]. Previous studies have found that cortical bone plays a major role in bone strength [15, 16]. But most studies focus on the microscopic level of cancellous bone, only few on the cortical bone [17].

It is a highly optimized bone structure with unified structure and function. Shipov et al. [18] found that elastic modulus in the cortical bone of OP group was less than normal group. Previous studies reported that changes in bone micro—structure is associated with the bone strength [19]. Thus, any change in micro—chemical composition, micro—structure and microscopic mechanical properties of cortical bone may affect the other and the macroscopic mechanical strength of bone.

Previous study has focused on the correlation of bone strength, micro—structure and micro—mechanics, founding that bone cortical porosity was significantly correlated with bone strength [20]. However, there are relatively few studies on the relationship of micro—structure, micro—mechanics and micro—chemical composition [20, 21]. The effects and weights of micro—chemical composition, micro—structure and micro—mechanical properties of cortical bone on bone strength remain unclear. Moreover, the sample size in previous study was relatively small (*n* = 28) [20]. Therefore, it is significant to study the role and weight of microscopic physical and chemical properties of femoral neck cortical bone on macroscopic mechanical strength during OP so as to prevent and treat osteoporotic fracture.

## Materials and methods

### Patients

Patients submitted to total hip joint replacement surgery at the General Hospital of Western Theater Command due to femoral neck fracture, femoral head necrosis, or hip arthritis were recruited from January 2018 to December 2020. The BMI, age, and gender were recorded. The inclusion criteria were as follows: a) total hip replacement surgery was performed; b) there were no obvious contraindications to surgery and no metabolic bone diseases (except OP); c) all patients had femoral neck BMD measured at the ipsilesional side; d) none had a history of psychiatric illness; e) patients or their families signed an informed consent. Exclusion criteria were as follows: a) patients who suffered from metabolic endocrine diseases (e.g., diabetes, thyroid diseases, hyperparathyroidism and rheumatoid arthritis); b) patients who received medicines affecting bone metabolism within 3 months prior to the study (e.g., glucocorticoids, thyroid drugs, vitamin D supplements and calcium supplements); c) patients had been prescribed antiosteoporotic treatment; d) severe organ failure; e) severe deformity at the measurement site; f) femoral neck samples were badly damaged during osteotomy.

### Measurement of BMD

Femoral neck BMD (g / cm2) from the ipsilesional side was obtained from DEXA scans in one week before surgery (Lunar prodigy, GE Medical Systems, Madison, WI, USA). Patients were grouped into normal group (T—score ≤ -1.0), osteopenia group (T—score <—1.0 and >—2.5), OP group (T—score ≤—2.5 or femoral neck fragile fracture) and severe OP group (fragility fracture with a T—score ≤—2.5) based on the results of femoral neck BMD according to the WHO diagnostic criteria [22] and the Guidelines for the diagnosis and management of primary osteoporosis (2017). During the total hip replacement surgery, osteotomy was initially operated 0.5—1.0 cm above the minor trochanter, and then performed under the femoral head to obtain the femoral neck samples. After eliminating surrounding soft tissues, the femoral neck was washed with phosphate buffered saline to remove blood and residues. Absorbing the excess water of samples with absorbent paper after collecting the cortical bone from the femoral neck region. Afterwards, the samples were kept in saline moistened gauze wraps and stored at -20 °C.

### Micro—CT imaging

The micro—structure of the cortical bone of femoral neck were scanned by Quantum GX Micro—CT (PerkinElmer Inc., Waltham, Massachusetts, United States). Cortical bone samples of femoral neck were placed in the sample holder vertically along the long axis. Then we filled some medical gauze strip around to prevent specimen from drifting during the scanning process. The scanning parameters used were as follows: voltage of 90 kV, current of 88 μA, scanning mode of 360° revolution, scanning time of 14 min, angle increment of 0.5, voxel size of 4.5 µm. Quantitative micro—CT analysis was performed with the accompanying software system (Analyze12.0). The analysis parameters include: cortical bone mineral density (cBMD), cortical bone thickness (Ct. Th) and cortical porosity (pore volume fraction) (Ct. Po).

### Nanoindentation assay

The elastic modulus and the hardness of the cortical bone of femoral neck were evaluated using a KEYSIGHT G200 nanoindenter (Keysight Technologies, California, United States). The samples were sequentially dehydrated once in 70%, 80%, 90% anhydrous ethanol and twice in 100% anhydrous ethanol, each phase for 24 h. Subsequently, the cortical bone tissues were infiltrated and embedded with a mixture of methyl methacrylate (MMA), dibutyl phthalate, and benzoyl peroxide. The surfaces were ground down using wet silicon–carbide papers P120, P600, P1200, P1500 and P4000 and polished with 1 mm and 0.3 mm Al_2_O_3_ suspensions used in combination with polishing cloths and the equipment PG—2DA (Guangmi Instruments Co., Ltd., Shanghai, China). Nanoindentation was performed in 10 randomly selected points along the cortical shell of the femoral neck under a microscope. We used a depth controlled approach with a fixed indentation depth of 1 µm. The testing parameters used were as follows: loading rate of 0.1 μN / s, unloading velocity of 0.1 mN / s and maximum displacement of 200 nm.

By knowing the geometry of indenter and depth of penetration, the area of contact is calculated, from which the elastic modulus and hardness were estimated.

### Compression tests

Tensile experiments were conducted by using MTS model 809 axial/torsional testing system (MTS Systems Corp., USA). The MTS testing machine was equipped by an axial hydraulic actuator that had a 200 kN axial capacity*.* The cortical bone samples of femoral neck were dissected and cut in approximately 5 mm height and the superior and inferior planes were sanded to be parallel to each other. Before testing, samples were placed in the testing system and preloaded with a static preload of -10 N for 30 s. Subsequently, the compression test was performed with a 0.02 mm/s speed until the appearance of obvious peak, and then the maximum load (L_max_) was automatically determined by the accompanying software MTS TestStar II (MTS Systems Corp., USA). During compression, the specimens were maintained at the room temperature of 23 ± 0.2 °C in a moist condition. Room temperature and humidity was monitored before, during, and after the experimental sessions to ensure that both remained stable.

### Fourier transform infrared spectroscopy (FTIR)

**Fig. 1:**
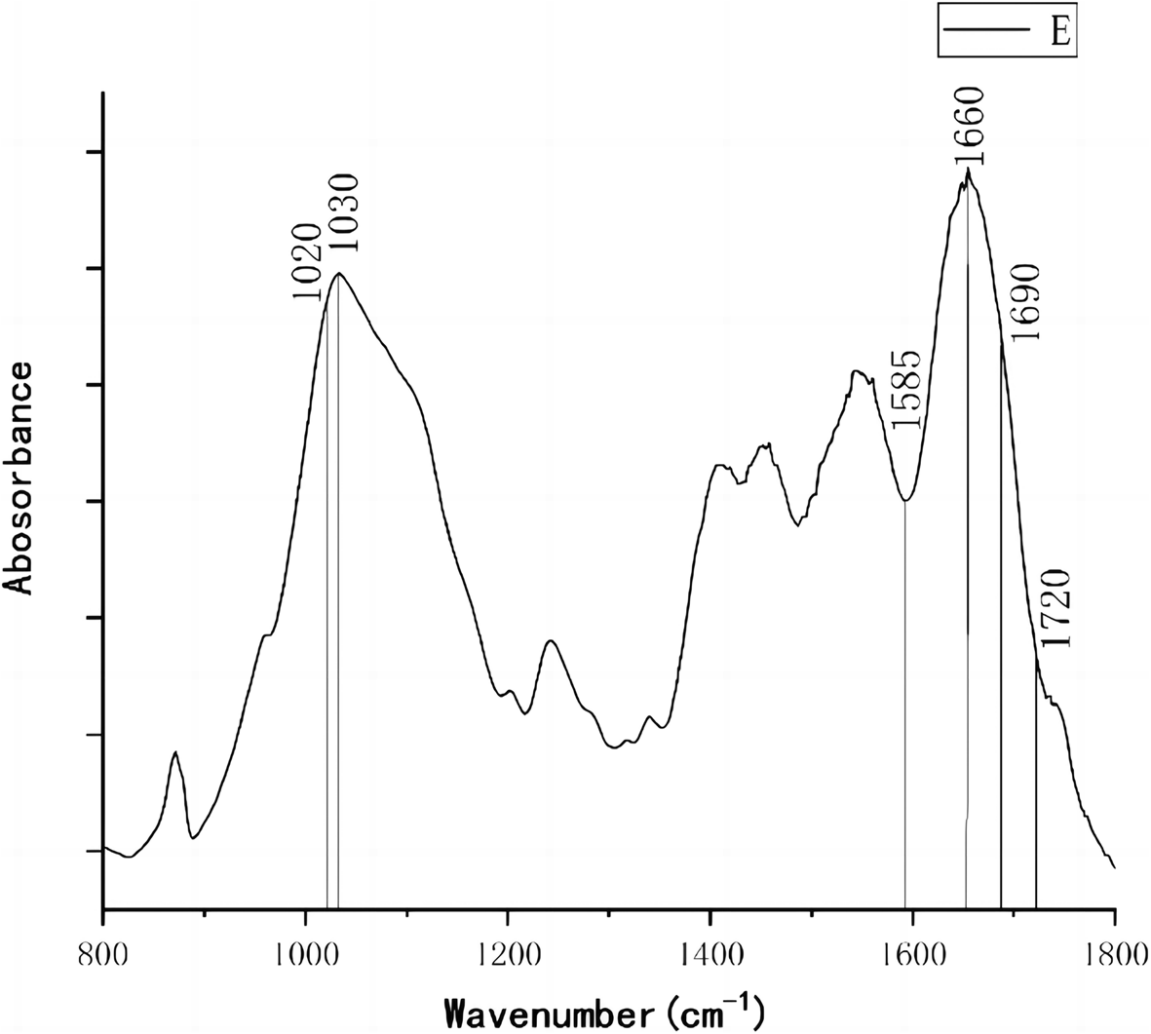
Infrared spectroscopy of femoral neck cortex

The cortical bone sample was dried and dehydrated in a 60 °C. The dried sample was then ground in an agate mortar. Potassium bromide (KBr, spectroscopic grade) powder was added next, and the powders were ground again until evenly blended. After the powders compressed into tablets on an infrared tablet press, fourier transform infrared spectroscopy (FTIR, Nicolet 5700, Thermo Fisher Scientific Inc., Waltham, MA, USA) was used to determine the chemical compositions of cortical bone. All the data were analyzed by using the OriginPro 2018C (OriginLab Corp., Northampton, MA, USA) (Fig. 1), and the indices examined included the mineral matrix ratio (the area under the phosphate peak (900—1200 cm-1) divided by the area under the amide I peak (1585—1720 cm-1)); mineral crystallinity (the ratio of relative peak height sub—bands at 1030 cm − 1 and 1020 cm-1 within the broad phosphate contour); collagen cross—linking ratio (indicative of the amount of nonreducible / reducible cross—linking) was determined by dividing the 1660 cm^−1^ band area by the 1690 cm^−1^ band area).

### X—ray diffraction tests

Cortical bone samples from the femoral neck were defatted, dehydrated, and dried in an oven at a constant temperature of 60 C. Afterwards, the dried samples were subjected to cryogenic grinding in a mortar and pestle. Then the dry powder samples were pipetted onto the zero—background slides and tested using X—ray diffraction (XRD) (PANalytical B.V, EMPYREAN, Almelo, Netherlands) with a Cu Kα radiation. The conditions were 40 kV and 100 mA, and the scan range was from 10 ^◦^ to 90 ^◦^. The crystal size of femoral neck cortical bone was analyzed using JADE 6.0 software (JADE, Materials Data Inc., Livermore, CA, USA). The Scherrer equation (Eq. (1)) is applied to calculate the crystal size. The terms of Eq. (1) are shape factor (κ) of 1, Cu Kα radiation average wavelength (λ) is 1.5418 Å, the full width at half maximum of the (200) peak (β) in radians, and the peak position divided by 2 of the (200) peak (θ) [23].

1 $$\uptau =\frac{\upkappa \bullet\uplambda }{\upbeta \bullet \mathrm{cos\theta }}$$

### Statistical analysis

For quantitative data with normal distribution and homogeneity of variance, one—way analysis of variance was performed, with differences among each group (normal group, osteopenia group, OP group and severe OP group) assessed using a Bonferroni post hoc test. The data conform to a normal distribution, but they are not conformable in the homogeneity of the variance was analysed by the non-parametric Kruskal—Wallis test for statistically significant differences. A chi-squared test was used for categorical variables. Pearson correlation coefficients was used to analyze the relationship of the femoral neck L_max_ and microscopic physical and chemical properties. The strength of the correlations was described by using the following classification: no correlation (0—0.29), weak correlation (0.30—0.59), moderate correlation (0.50—0.69), strong correlation (0.70—0.89), or very strong correlation (0.90—1.0). Multivariate linear regression analyses were performed to identify significant factors that affected the femoral neck L_max_. *P* < 0.05 indicated that the difference was statistically significant. All statistical analyses were performed with SPSS 25.0 software (SPSS Inc., IL, USA).

## Results

### General information of the patients

A total of 115 patients were selected in this study with an average age of 61.17 ± 10.72 years and an average BMI of 24.50 ± 3.48 kg / cm^2^. Patients in the severe OP were significantly older than normal and osteopenia group (*P* < 0.01). The osteopenia and the osteoporosis groups both had a significantly older age than normal group (*P* < 0.01). There were no significant differences in age among other groups (*P* > 0.05). The femoral neck T—score in the severe OP group was significantly lower than those in the normal, osteopenia and OP groups (*P* < 0.0001). The femoral neck T—score in OP and osteopenia groups were both significantly lower compared with the normal group (*P* < 0.05). No differences were observed between the osteopenia group and the OP group (*P* > 0.05). The femoral neck BMD gradually decreased during osteoporosis, the difference between groups was significant (*P* < 0.01). There were no significant difference in age and sex between groups (*P* > 0.05) (Table 1).

**Table 1:** Comparison of general clinical information of the patients in each group

Parameters	Normal ^a^ (*n* = 27, fragility fracture n = 0)	Osteopenia ^b^ (*n* = 32, fragility fracture *n* = 0)	Osteoporosis ^c^ (*n* = 29, fragility fracture n = 17)	Severe osteoporosis ^d^ (*n* = 27, fragility fracture n = 27)	*x*^*2/*^*F/H*	*P*-value
Gender (male/female)	6/21	7/25	6/23	5/22	0.140	0.987
Age (years)	51.48 ± 10.71	60.38 ± 6.37^*^	63.52 ± 9.01^*^	69.30 ± 8.97^*#^	19.342	< 0.0001
BMI (kg/cm^2^)	24.85 ± 3.57	24.58 ± 4.06	24.83 ± 2.8	23.72 ± 3.37	0.631	0.596
Femoral neck BMD (g/cm^2^)	0.94 ± 0.12	0.84 ± 0.14^*^	0.76 ± 0.09^*#^	0.61 ± 0.1^*#▲^	38.528	< 0.0001
Femoral neck T-score	0.00 ± 0.90	1.54 ± 0.51^*^	-1.78 ± 0.76^*^	-2.91 ± 0.40^*#▲^	82.111	< 0.0001

Data are presented as the mean ± SD for age, BMI, femoral neck BMD, femoral neck T-score and femoral neck L_max_

*BMI* body mass index, *BMD* bone mineral density

^a^ T-score ≥ -1.0,

^b^ T-score < -1.0 and > -2.5,

^c^ At least one of the following criteria fulfilled: (a) T-score ≤ -2.5; (b) femoral neck fragile fracture),

^d^ Fragility fracture with a T-score ≤ -2.5

^*^ *P* < 0.05, compared with normal group

^#^ *P* < 0.05, compared with osteopenia group

^▲^ *P* < 0.05, compared with osteoporosis group

### Structural analysis

**Fig. 2:**
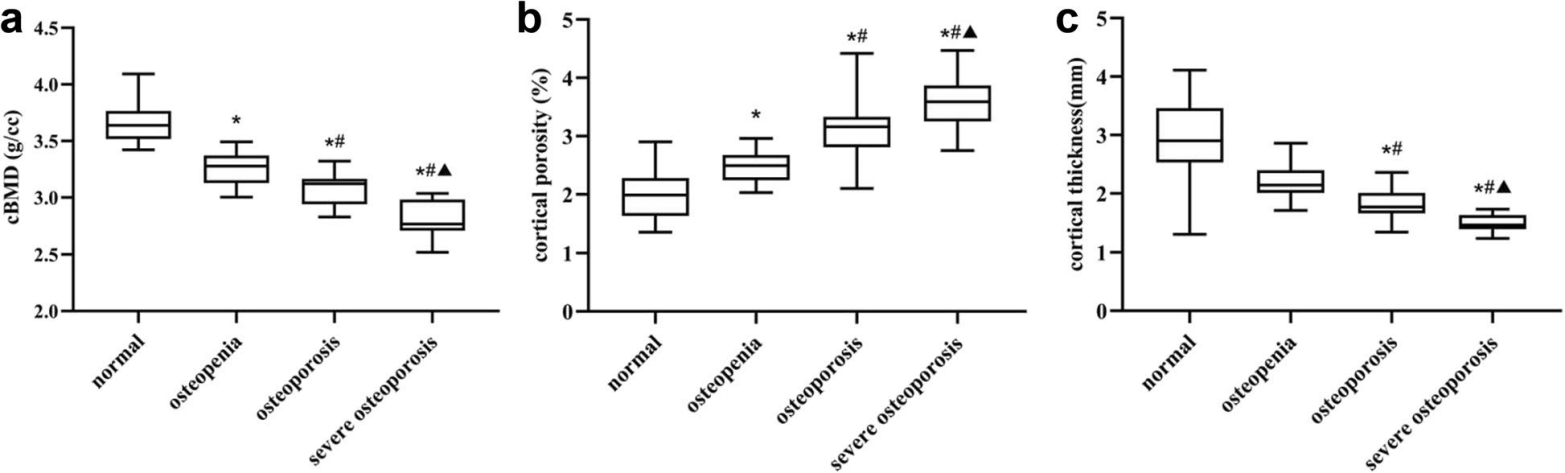
The change in femoral neck micro-structure over the different groups. **A** Comparison of cBMD among different groups; **B** Comparison of Ct. Po among different groups; **C** Comparison of Ct. Th among different groups. * *P* < 0.05, compared with normal group; # *P* < 0.05, compared with osteopenia group; ^▲^ *P* < 0.05, compared with osteoporosis group

Micro—CT data from the cortical bone are shown in Fig. 2. It can be seen from the figures that femoral neck cBMD gradually decreased, whereas Ct. Po gradually increased. The differences among the groups were significant (*P *< 0.001). The femoral neck Ct. Th showed a gradual downward trend, but the changes between the normal group and the osteopenia group were not different (*P* = 0.058). The differences were statistically significant for the remaining groups (*P* < 0.05).

### Nanoindentation tests

**Fig. 3:**
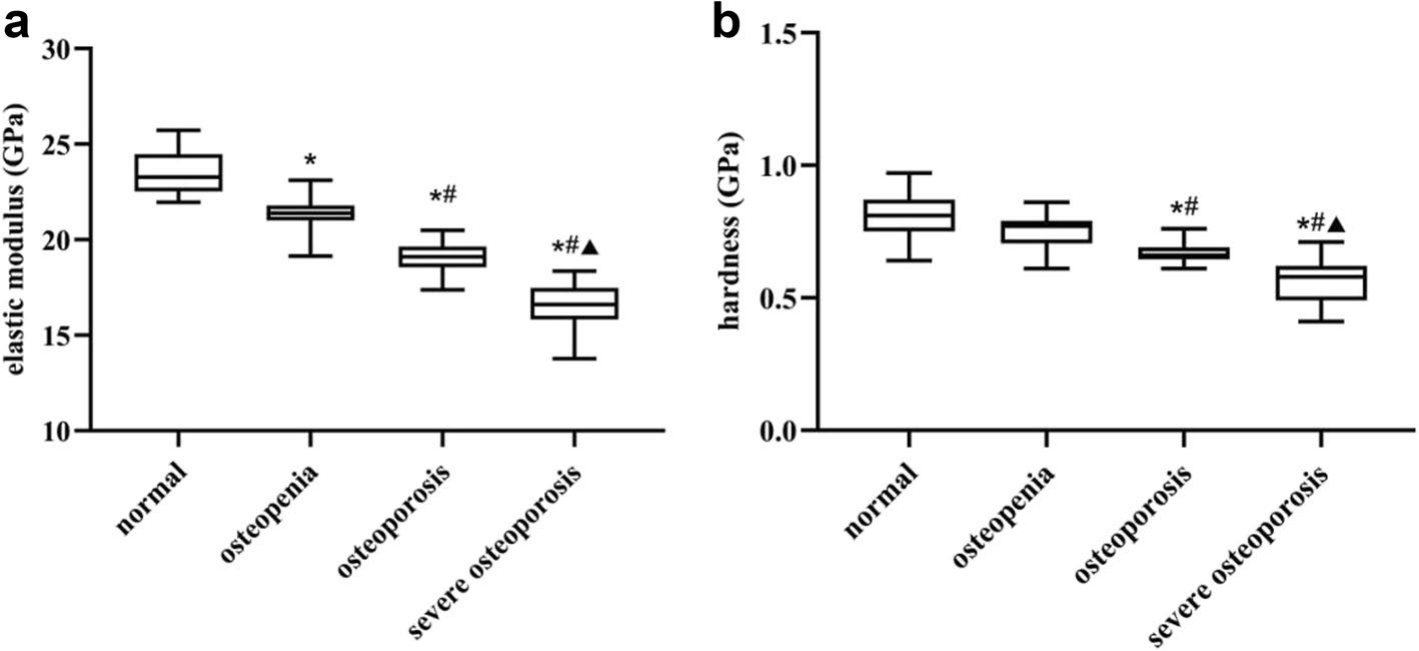
The change in femoral neck micro-mechanical properties over the different groups. **A** Comparison of elastic modulus among different groups; **B** Comparison of hardness among different groups. ^*^ *P* < 0.05, compared with normal group; ^#^ *P* < 0.05, compared with osteopenia group; ^▲^ *P* < 0.05, compared with osteoporosis group

The mechanical indices (elastic modulus and hardness) of cortical bones from femoral neck were examined by nanoindentation. As shown in Fig. 3A, the cortical elastic modulus of femoral neck was decreased significantly and progressively across groups. Differences between all groups were statistically significant (*P* < 0.01). It can be seen from the Fig. 3B. the femoral neck hardness revealed a gradually decreasing trend. However, the changes between the normal group and the osteopenia group were not different (*P* = 0.777). The differences were statistically significant for the remaining groups (*P* < 0.05).

### *Compression* *test*

**Fig. 4:**
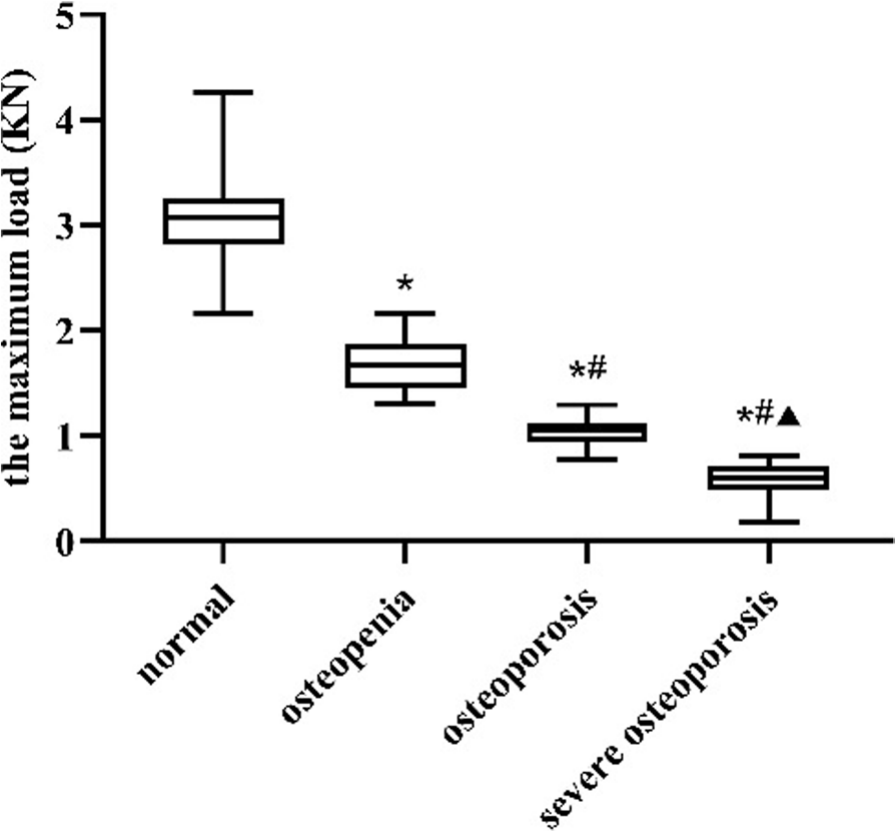
The change in femoral neck maximum load over the different groups. ^*^ *P* < 0.05, compared with normal group; ^#^ *P* < 0.05, compared with osteopenia group; ^▲^ *P* < 0.05, compared with osteoporosis group

The results of the compression test are presented in Fig. 4. The femoral neck L_max_ in the severe OP group was significantly lower than those in the normal, osteopenia and OP groups (*P* < 0.0001). The femoral neck L_max_ in the OP groups was significantly lower than those in the normal and osteopenia groups. Compared with the normal group, the femoral neck L_max_ in the osteopenia group was significantly decreased. The comparisons among all the groups were statistically significant (*P* < 0.0001). The femoral neck L_max_ showed a 46.48% decrease in the osteopenia group compared with the normal group. Similarly, the femoral neck L_max_ of osteoporosis group decreased 36.94% compared with that of osteopenia group. Compared with the osteoporosis group, the femoral neck L_max_ in the severe osteoporosis group decreased by 44.39%.

### Fourier-transform infrared (FTIR) results

**Fig. 5:**
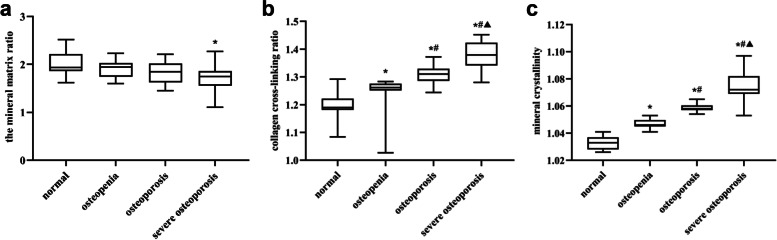
The change in femoral neck micro-chemical composition over the different groups. **A** Comparison of matrix ratio among different groups; **B** Comparison of cross—linking ratio among different groups; **C** Comparison of mineral crystallinity among different groups. * *P* < 0.05, compared with normal group; # *P* < 0.05, compared with osteopenia group; ^▲^ *P* < 0.05, compared with osteoporosis group

Results of FTIR analysis on femoral neck cortical bone specimens are shown in Fig. 5. The femoral neck mineral matrix ratio in the severe OP group was significantly lower than those in the normal group (*P* = 0.002). There was no significant difference among the rest of the groups (*P* > 0.05). With the bone loss of femoral neck, both collagen cross-linking ratio and mineral crystallinity increase gradually. The differences among the groups were all significant (*P* < 0.05).

### XRD analysis

**Fig. 6:**
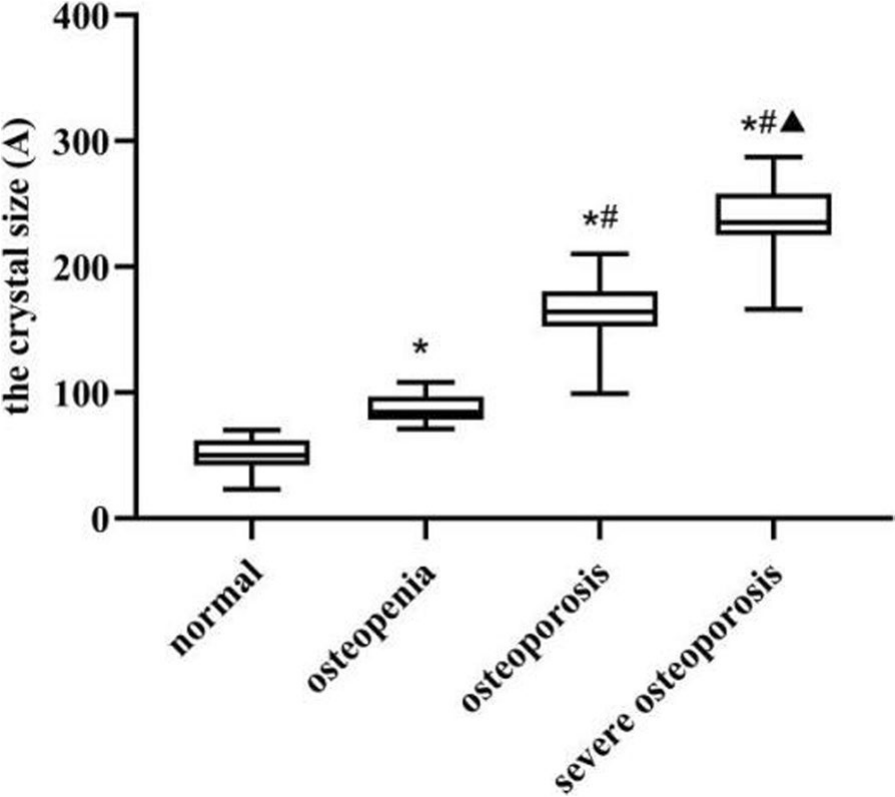
The change in femoral neck crystal size over the different groups. * *P* < 0.05, compared with normal group; # *P* < 0.05, compared with osteopenia group; ▲ *P* < 0.05, compared with osteoporosis group

The XRD analysis is shown in Fig. 6. During the progression of OP, the crystal size of femoral neck cortical bone gradually rose in all groups, and all differences were statistically significant (*P* < 0.05).

### Correlation of the femoral neck microscopic parameters

Pearson correlation analysis results showed that there were significant correlations of microscopic physical and chemical properties of femoral neck (*P* < 0.001). Among them, the crystal size of femoral neck cortical bone was strongly correlated with the elastic modulus, hardness, *cBMD, Ct. Po, Ct. Th, collagen cross—linking* ratio and mineral crystallinity (r > 0.7), whereas moderately correlated with the mineral matrix ratio (r > 0.4). The elastic modulus of femoral neck cortical bone was strongly correlated with the hardness, cBMD, Ct. Po, Ct. Th, collagen cross-linking ratio and mineral crystallinity (*r* > 0.7), whereas moderately correlated with the mineral matrix ratio (*r* > 0.4). The hardness of femoral neck cortical bone was strongly correlated with the Ct. Po and mineral crystallinity (r > 0.7), whereas moderately correlated with the cBMD, Ct. Th, mineral matrix ratio and collagen cross—linking ratio (r > 0.4). The cBMD presented a strong correlation with the Ct. Po, Ct. Th, collagen cross—linking ratio and mineral crystallinity (r > 0.7), with weak correlation with the mineral matrix ratio (r < 0.4). The Ct. Th of femoral neck cortical bone had strong correlation with the Ct. Po and mineral crystallinity (r > 0.7), moderate correlation with the collagen cross-linking ratio (r > 0.4), and weak correlation with the mineral matrix ratio (r < 0.4). The Ct. Po of femoral neck cortical bone was strongly correlated with the mineral crystallinity (*r* > 0.7), moderately correlated with the collagen cross-linking ratio (*r* > 0.4), and weakly correlated with the mineral matrix ratio (*r* < 0.4). The mineral matrix ratio presented a moderate correlation with the collagen cross—linking ratio and mineral crystallinity (*r* > 0.4). Finally, the collagen cross—linking ratio of femoral neck cortical bone had strong correlation with mineral crystallinity (*r* > 0.7) (Table 2).

**Table 2:** Pearson correlation coefficients of femoral neck microscopic parameters

Parameters		crystal size	elastic modulus	hardness	cBMD	Ct.Th	Ct.Po	mineral -matrix ratio	mineral crystallinity
elastic modulus	r	-0.955							
hardness	r	-0.845	0.870						
cBMD	r	-0.811	0.827	0.695					
Ct. Th	r	-0.763	0.802	0.658	0.706				
Ct. Po	r	0.817	-0.854	-0.740	-0.714	-0.753			
mineral matrix ratio	r	-0.421	0.470	0.415	0.330	0.324	-0.382		
mineral crystallinity	r	0.910	-0.916	-0.760	-0.802	-0.738	0.772	-0.409	
collagen cross—linking ratio	r	0.803	-0.815	-0.664	-0.712	-0.684	0.683	-0.444	0.786

*cBMD* cortical bone mineral density, *Ct. Th* cortical bone thickness, *Ct. Po* cortical porosity

### *Correlation of the femoral neck microscopic parameters with femoral neck L*_*max*_

As shown in Table 3, the femoral neck L_max_ was significantly correlated with all femoral neck microscopic parameters (*P* < 0.001). Besides, the femoral neck L_max_ was strongly correlated with the crystal size, elastic modulus, hardness, cBMD, total porosity, collagen cross—linking ratio, mineral salt crystallinity and the Ct. Th (*r* > 0.7), whereas moderately correlated with the mineral matrix ratio (*r* > 0.4). Among the four indicators of micro—chemical composition, we found that the femoral neck L_max_ was strongest correlated with the crystal size (*r* = -0.880, *P* = 0.000). Compared with the other three indicators of the femoral neck micro-structure, the femoral neck L_max_ was strongest correlated with the cBMD (*r* = 0.855, *P* = 0.000). Finally, we found that the elastic modulus of femoral neck was strongest correlated with the femoral neck L_max_ compared with the femoral neck hardness (*r* = 0.936, *P* = 0.000).

**Table 3:** Pearson correlation coefficients of femoral neck L_max_ and other parameters

Parameters	Femoral neck L_max_ *r P*	
crystal size	-0.880	0.000
elastic modulus	0.936	0.000
hardness	0.783	0.000
cBMD	0.855	0.000
Ct. Th	0. 829	0.000
Ct. Po	-0.814	0.000
mineral matrix ratio	0.439	0.000
mineral crystallinity	-0.877	0.000
collagen cross—linking ratio	-0.784	0.000

*cBMD* cortical bone mineral density, *Ct. Th* cortical bone thickness, *Ct. Po* cortical porosity

The femoral neck L_max_ were used as dependent variable, and femoral neck elastic modulus, crystal size, cBMD, age, BMI and gender were all included as independent variables in multivariate linear regression models. There was no interaction among variables presented in Table 4 was found in multiple linear regression analyses. Multivariate linear regression analysis revealed that the femoral neck L_max_ was positively correlated with the femoral neck elastic modulus (*β* = 0.920, *P* = 0.000), crystal size (*β* = 0.226, *P* = 0.028), and cBMD (*β* = 0.269, *P* = 0.000). In addition, we found that femoral neck L_max_ was strongest correlated with the femoral neck elastic modulus (Table 4).

**Table 4:** Multiple linear regression analysis between femoral neck L_max_ and microscopic parameters

Parameters	Standardized Coefficients Beta	*P*—value
crystal size	0.226	0.028
elastic modulus	0.920	0.000
cBMD	0.269	0.000
Adjusted—R^2^	0.899	

Adjusted for age, BMI and sex cBMD cortical bone mineral density

## Discussion

With an increasing aging population, the incidence of femoral neck fractures in the middle and elderly is increasing year by year [24, 25]. Due to the high rate of morbidity and mortality, femoral neck fragility fractures have received more and more attention [25]. In our study, we found femoral neck micro—structure was changed, as well as micro—mechanical properties and the load at breaking were decreased during the progression of OP. Also, we found the change of the relationship between collagen and mineral with the bone loss. These above changes suggest that the macroscopic and microscopic properties of the femoral neck change progressively with the progression of OP.

Femoral neck L_max_ is defined as the maximum force that femoral neck can tolerate before fracture, and it is an important parameter in regard of bone mechanical integrity [26]. In our study, the largest decline in femoral neck L_max_ (46.48%) was observed between normal group and osteopenia group, which may indicate that the strength of the femoral neck begins to decrease at the transitional stage of osteoporosis (i.e., osteopenia). This may also explain the clinical occurrence of fragility fractures in some patients as the BMD >—2.5 SD [27, 28].

Cortical bone plays an important role in the mechanical properties and fracture risk. In addition to cBMD, other cortical bone characteristics can also contribute to the fracture resistance of entire skeleton. Among these, Ct. Th and cortical cross—sectional area are most often used as the surrogate for mechanical properties of cortical bone [29]. However, the micro—structural properties such as cortical bone porosity are also relevant to the bone macroscopic mechanical properties [29]. Abraham et al. [30] found that there is a significant correlation between Ct. Po and bone strength. Our study showed that the femoral neck cortical bone showed a gradual degeneration of the micro—structure and L_max_ from normal bone mass to severe OP. The above results suggested that with the cortical bone loss, the decrease of cBMD, thinning of cortical bone as well as the increase of cortical porosity may further lead to the weakening of bone macro—mechanical properties and increased risks of fracture.

In our experiment, we observed that with the progression of OP, the nanoindentation modulus and hardness of femoral neck showed a gradual decrease as well as the femoral neck L_max_ also showed a decreasing trend. Further study showed that there was a positive correlation between cortical bone micro—mechanics and L_max_. This agrees with the findings of Jeffry et al. [31] concluded that the changes in micro—mechanical properties are associated with risk of fracture, and the changes in the relationship between micro—mechanical properties and chemical composition cause an increased risk of fracture. It can be inferred that OP can affect the micro—mechanical properties of femoral neck cortical bone, which will further affect the L_max_.

In our survey, the mineral matrix ratio of femoral neck cortical bone decreased gradually from normal bone mass to severe osteoporosis. These suggest that the bone loss of femoral neck may be associated with the imbalance in the ratio between bone collagen and mineral, which may also affect bone strength in femoral neck [32]. Correlation analysis revealed that there is a significant relationship between the mineral matrix ratio and the L_max_ of the femoral neck cortical bone, which further illustrated that the imbalance in the ratio between bone collagen and mineral lead to the changes of femoral neck macroscopic mechanical properties.

Bone mineral crystallinity is related to the crystal size and crystal quality, and the crystal size can affect the bone tissue material properties [33]. We found that the mineral crystallinity of femoral neck cortical bone gradually increased while the micro—mechanical properties gradually decreased during the process of bone loss, and there was a significant relationship of them. Gourion—Arsiquaud et al. [34] mentioned that bone mineral crystallinity is associated with fracture risk. They speculate that the larger bone mineral crystals may be more brittle and weaker. Also, the larger crystals may not be able to align as well with the collagen matrix, which attenuated the interaction between the crystals and the collagen matrix, thus leading to a reduction of the bone mechanical properties and an increase of fracture risk.

In our study, we found that the femoral neck cortical collagen cross-linking ratio gradually increased from normal bone mass to severe osteoporosis. There was a significant correlation between collagen cross—linking ratio, elastic modulus, hardness as well as bone load at breaking. This suggested that collagen cross-linking increases with the progression of OP, which severely limiting the deformation of collagen fibrils, further decreasing bone toughness, ductility and mechanical strength [35]. These findings suggested that the bone collagen cross-linking can be further amplified from affecting bone tissue at the microscopic level to affecting mechanical properties of bone tissue at the macroscopic level. Thus, higher collagen cross—linking ratios of cortical bone are associated with increased fracture risk [36].

The femoral neck is situated in the articular capsule of the hip, in normal gait, the greatest stresses occur in the sub—capital and mid-femoral neck region, where maximum compressive stresses occur inferiorly [37]. In response to this mechanical need, the femoral neck cross-section is oval in shape, with the cortical bone forming a ring—shaped shell and the interior filled with cancellous bone to ensure the overall bone mechanical properties of the femoral neck [38, 39]. The femoral neck was made of 80% cortical bone and 20% trabecular bone, thus the cortical bone has been reported to be the main determinant of the femoral neck bone strength [40]. Existing studies also support this conclusion. In human cadaver femurs, cortical bone has been reported to be the main determinant of the femoral neck bone strength, while trabecular bone only contributes marginally to bone strength at this site [41]. Zebaze et al. [42] found that the cortical porosity for populations over 65 years of age was significantly higher than those between ages 50 and 80 years, resulting in thinner cortical bones. All the above results suggests that in osteoporotic patients, the femoral neck cortical bone matrix (mineral and collagen) is extensively lost and disordered, resulting in the cortical thinning, the cortical trabecularization, and the enlargement of the bone marrow cavity, thus causing a significant decrease in the overall mechanical strength of the femoral neck and an increased risk of fracture.

Our experiment also has some limitations. Firstly, all specimens were dehydrated in graded anhydrous ethanol in the nanoindentation experiments, the state of bone specimens is quite different compared to the physiological state of bone tissues. Besides, our study is a single ‐ center study, the representativeness of the sample might be limited. In the future, we plan to conduct a multicenter case control study to overcome the limitations of the current study.

## Conclusions

In summary, the microscopic physical and chemical properties and macroscopic mechanical properties of the femoral neck cortical bone can influence each other. Moreover, at the microscopic scale, the micro—structure, micro—mechanical properties and micro—chemical composition are interact with each other, and together maintain the femoral neck L_max_. In the progression of OP, the quantitative and qualitative changes of microscopic physical and chemical properties all can affect femoral neck L_max_. Among them, the elastic modulus has the most significant effect on femoral neck L_max_.

## Data Availability

The datasets used and analyzed during the current study are available from the corresponding author Da Liu on reasonable request.

## Abbreviations

ANOVA: Analysis of variance
BMD: Bone mineral density
BMI: Body mass index
cBMD: Cortical bone mineral density
Ct. Th: Cortical bone thickness
Ct. Po: Cortical porosity
DEXA: Dual energy X—ray absorptiometry
OP: Osteoporosis
